# Virulence and resistance gene analysis of Rothia nasimurium by whole gene sequencing

**DOI:** 10.1038/s41598-025-95405-z

**Published:** 2025-03-27

**Authors:** Ziyue Lu, Sun He, Ali Adnan, Wenyu Fan, Jinliang Sheng, Yanming Sun, Yanbing Zhang, Gang Wang

**Affiliations:** 1 College of Animal Science and Technology, Shihezi University, Shihezi, Xinjiang China; 2 TECON Pharmaceutical Co.,Ltd, Urumqi, 830011 China

**Keywords:** *Rothia*, *Rothia nasimurium*, Draft genome sequencing, Resistance genes, Virulence genes, Pathogenicity, Sheep, Computational biology and bioinformatics, Genetics, Microbiology

## Abstract

A batch of sheep in a sheep farm in Xinjiang, China, died suddenly; a bacterial strain was isolated from the abdominal fluid of the sick and dead sheep, and identified as *Rothia nasimurium* by 16S sequencing, and the strain Y1 was subjected to drug sensitivity test with Draft gene sequencing. The results of the drug sensitivity test revealed the strain’s resistance to 9 antibiotics, with sensitivity exhibited solely towards amikacin and vancomycin. Phylogenetic tree analysis confirmed that it was related to *Rothia nasimurium strain E1706032* and *Rothia sp.SD9660Na*. The draft genome sequencing results showed that the total length of the gene was 2,387,685 bp, and the GC content was 59.35%. VFDB database analysis identified 112 annotated genes in Y1, including those related to bacterial adhesion, regulation, nutrient metabolism factors, hemolysin, immunomodulation, and iron uptake proteins. CARD database analysis showed that Y1 was resistant to a variety of antibiotics such as glycopeptides, tetracyclines, aminoglycosides and polypeptides. Animal pathogenicity tests have shown that Y1 can cause lung damage, coat loss and skin inflammation. This study revealed a series of virulence and drug resistance genes and pathogenicity of Y1. The results of this study have important reference value for prevention and treatment of *Rothia* infection in the future.

## Introduction

*Rothia nasimurium,* a gram-positive facultative anaerobic coccus within the Micrococcaceae family, was first identified by M.D. Collins et al. in 2000, who discovered it in the nasal cavity of rats^1^. *Rothia nasimurium* belongs to the *Rothia spp*. *Rothia* has been commonly detected in the oral^2^ and intestinal tracts of humans^3^, as well as in pigs and rodents, where it is considered part of the normal flora. In 2011, Nan Li et al. isolated a strain of *Rothia nasimurium* from the air of a ranch, indicating its potential airborne nature^4^. Subsequently, in 2014, Bemis et al. isolated a strain from various sources, including skin lesions, tonsils, the external ear canal, and semen of dogs. Their findings demonstrated strong synergistic hemolytic effects with *Staphylococcus aureus* in primary culture, highlighting *Rothia nasimurium* as a conditionally pathogenic bacterial strain^5^. The primary clinical approach to bacterial infections in animals involves the use of antibiotic drugs, yet the escalating issue of bacterial resistance has become a significant public health concern^6^. In 2021, Wang, M et al. isolated a strain of *Rothia nasimurium E1706032* from ducks, revealing extensive antibiotic resistance and the potential for transmission via mobile units^7^, this raises concerns about *Rothia nasimurium*'s airborne nature, posing a risk for the transmission of challenging infections between humans and animals.

Currently, *Rothia nasimurium* has been successfully isolated from diverse animal samples, including dogs, rabbits, pigs, geese, and ducks. However, there is a limited amount of information available on the virulence genes and drug resistance genes associated with *Rothia nasimurium*. The comprehension of gene metabolic pathways, protein sequences remain inadequate. Additionally, the infection route, pathogenic mechanisms, as well as clinical treatment and prevention strategies for this bacterium necessitate further exploration.

In a recent occurrence, a sudden outbreak of various diseases, such as depression and loss of appetite, affected several sheep in a Xinjiang sheep farm, resulting in fatalities. This study involved the collection and culturing of peritoneal effusion from the deceased sheep to isolate and identify a strain of *Rothia nasimurium*. The draft genome of the isolated strain was subsequently sequenced using illumina’s second-generation sequencing technology. Based on the sequencing results, a phylogenetic tree was constructed using housekeeping genes, and an analysis of their protein functions, gene metabolic pathways, drug resistance genes, and virulence genes was conducted. This comprehensive analysis aims to provide a theoretical foundation for future strategies in treating and preventing clinical symptoms caused by this bacterium.

## Results

### Strain identification and phylogenetic tree analysis results

**Fig. 1:**
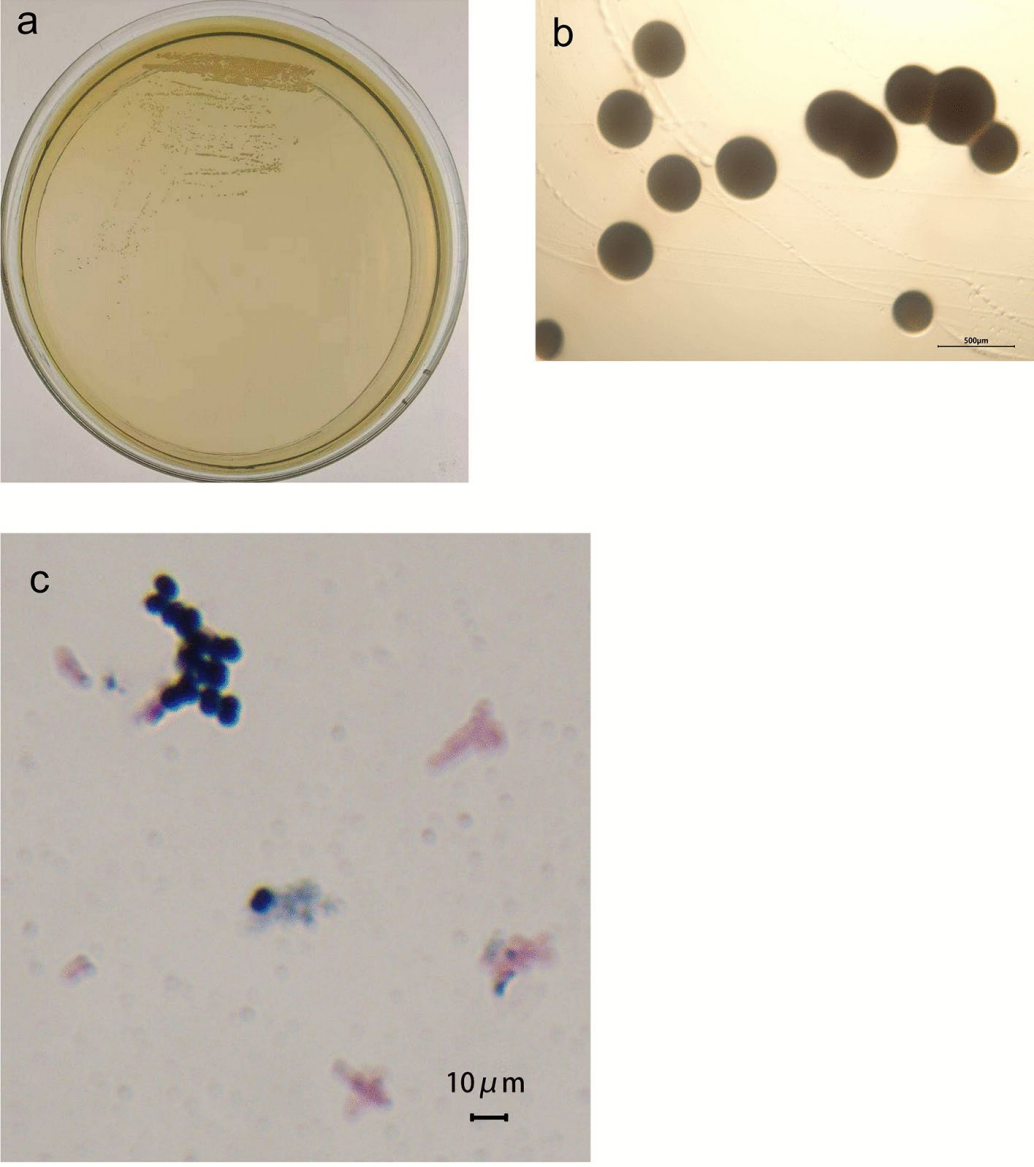
Purified bacterial colonies cultured on, Tryptose Soya Agar medium, Colony morphology under 40 × light microscope, Gram staining of the isolated bacteria (1000 × magnification).

Abdominal fluid was inoculated into Trypticase Soy Broth medium, Isolation of strains in Tryptose Soya Agar medium by incision method. *Staphylococcus* *aureus*, *Escherichia coli*, and *Streptococcus* account were identified as the major organisms, one strain, differed from the rest of the colony, formed smooth grayish-white raised colonies less than 0.5 mm in diameter in tryptic soy agar medium (Fig. 1 a and b). The bacteria were observed as Gram-positive cocci with a diameter greater than 1.0 μm under a 100 × oil microscope (Fig. 1 c) , this strain was named Y1.

The agarose gel electrophoresis revealed a target band with a size of 1492 base pairs (Supplementary Fig. 1 and Fig. 5). Subsequent 16S sequencing confirmed the identity of the isolate as *Rothia nasimurium*. Various levels of Rothia nasimurium has detected in the nasal cavity of different healthy sheep (Supplementary Fig. 4).

**Fig. 2:**
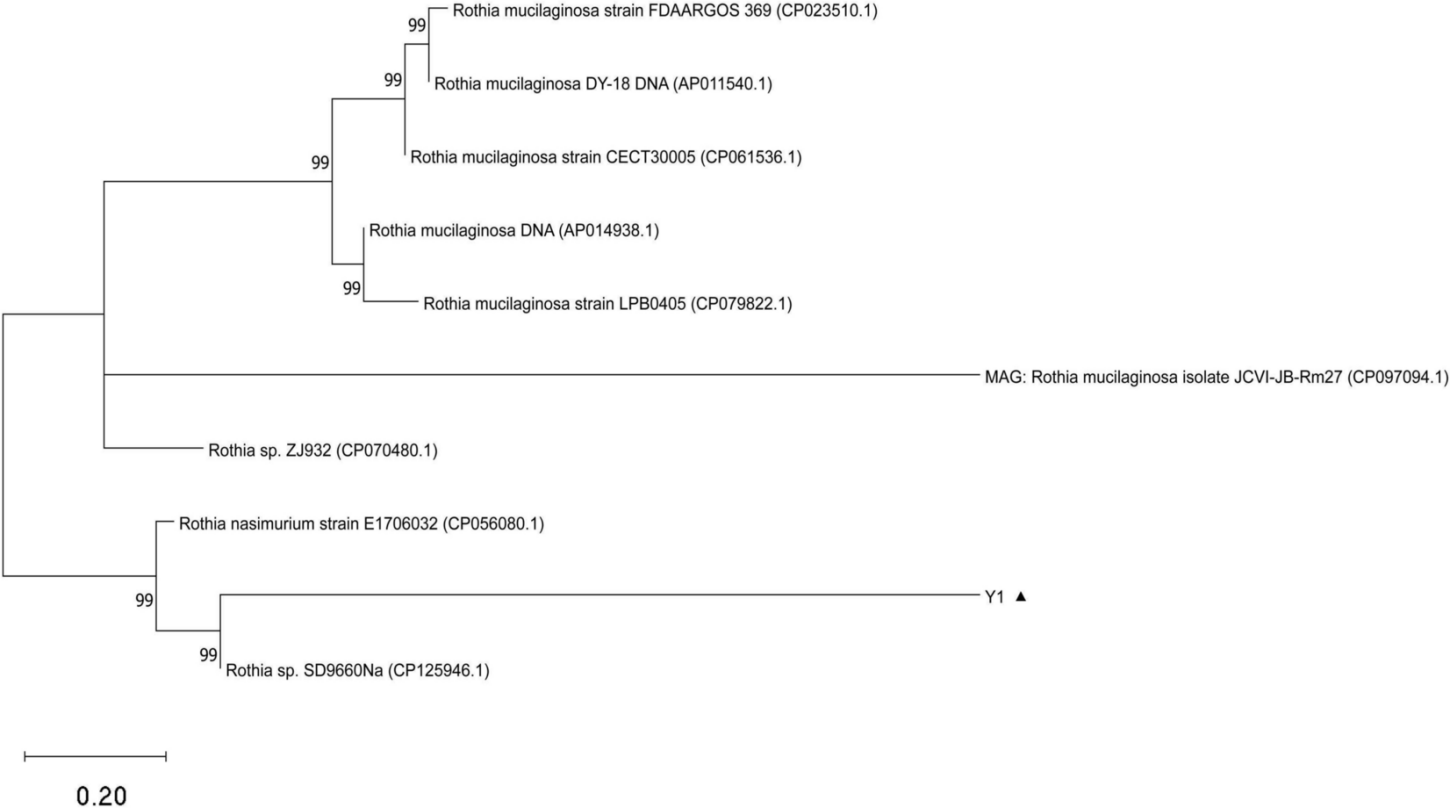
illustrates the construction of a phylogenetic tree based on the housekeeping genes in *Rothia nasimurium* Y1, ▲The strain Y1 isolated in this study. The phylogenetic tree, constructed using multiple housekeeping genes, provides a clear representation of the genetic relationships of *Rothia nasimurium* Y1, indicating its closest affinity to *Rothia nasimurium* strain E1706032 and Rothia sp. SD9660Na.

Housekeeping genes (adk, atpG, frdB, mdh, zwf) of *Rothia* were identified through the MLST website, and the corresponding gene sequences were obtained. Phylogenetic trees were then constructed using MEGA software(v11,0) (https://www.megasoftware.net), after comparing these sequences with those of 10 reference strains from the NCBI database through BLAST (Fig. 2). The analysis of the phylogenetic tree revealed that the current isolate, strain *Y1*, shares the closest relationship with *Rothia nasimurium strain E1706032*, which was isolated from the brain of a duck in Shandong, China, in 2017. Additionally, *Y1* is closely related to *Rothia sp. SD9660Na*, which was isolated from the nasal cavity of a dog in Switzerland in 2007. The results are consistent with those of FastANI software(v1.0) ( https://github.com/ParBLiSS/FastANI/releases) (Table 1).

**Table 1:** Phylogenetic tree *Rothia strain information.*

Strain	ANI	Year	Host	Country
Rothia nasimurium strain E1706032	92.28	2017	Duck	China ShanDong
Rothia sp. SD9660Na	91.53	2007	Canis lupus	Switzerland
Rothia mucilaginosa strain: NUM-Rm6536	74.51	2014	human	Japan: Osaka
Rothia mucilaginosa strain CECT30005	74.26	2017	Homo sapiens	Spain: Valencia
Rothia mucilaginosa strain LPB0405	74.26	2020	Human sputum	South Korea
Rothia mucilaginosa strain FDAARGOS_369	74.04	2014	Homo sapiens	USA: DC
Rothia mucilaginosa isolate JCVI-JB-Rm27	74.26	2017	human saliva	USA
Rothia mucilaginosa DY-18 DNA	73.54	2010	human	Japan
Rothia sp. ZJ932	73.35	2018	marmot	China

### Drug sensitivity test results

The results of the drug sensitivity test indicated that out of the 11 antibiotics commonly used in clinical practice, the isolated strain *Y1* exhibited sensitivity only to amikacin (aminoglycoside), vancomycin (glycopeptide). cefazolin (cephalosporin), norfloxacin (III quinolones), cotrimoxazole (sulfonamide), ampicillin (β-lactam), meropenem (β-lactam), tetracycline (tetracycline), clindamycin (Lincomycin ), azithromycin (macrolide) and florfenicol (chloromycetin) were all resistant (Table 2) . These findings suggest that the strain demonstrates multiple drug resistance.

**Table 2:** Results of the drug sensitivity test.

Antibiotics category	Drug names	Judging Standard	Inhibition zone diameters (mm)	Results
aminoglycoside	amikacin	≥ 17, ≤ 14	23	sensitive
Glycopeptide	vancomycin	≥ 21, ≤ 17	22	sensitive
III quinolones	norfloxacin	≥ 17, ≤ 12	0	resistant
sulfonamide	Cotrimoxazole	≥ 18, ≤ 13	6	resistant
β-lactam	ampicillin	≥ 15, ≤ 11	14	resistant
	meropenem	≥ 20, ≤ 15	16	resistant
cephalosporin	cefazolin	≥ 23, ≤ 19	5	resistant
tetracycline	Tetracycline	≥ 15, ≤ 11	0	resistant
lincomycin	clindamycin	≥ 21, ≤ 14	2	resistant
macrolide	azithromycin	≥ 13, ≤ 12	0	resistant
chloromycetin	florfenicol	≥ 18, ≤ 12	3	resistant

### Genomic analysis of* Rothia nasimurium*

**Fig. 3:**
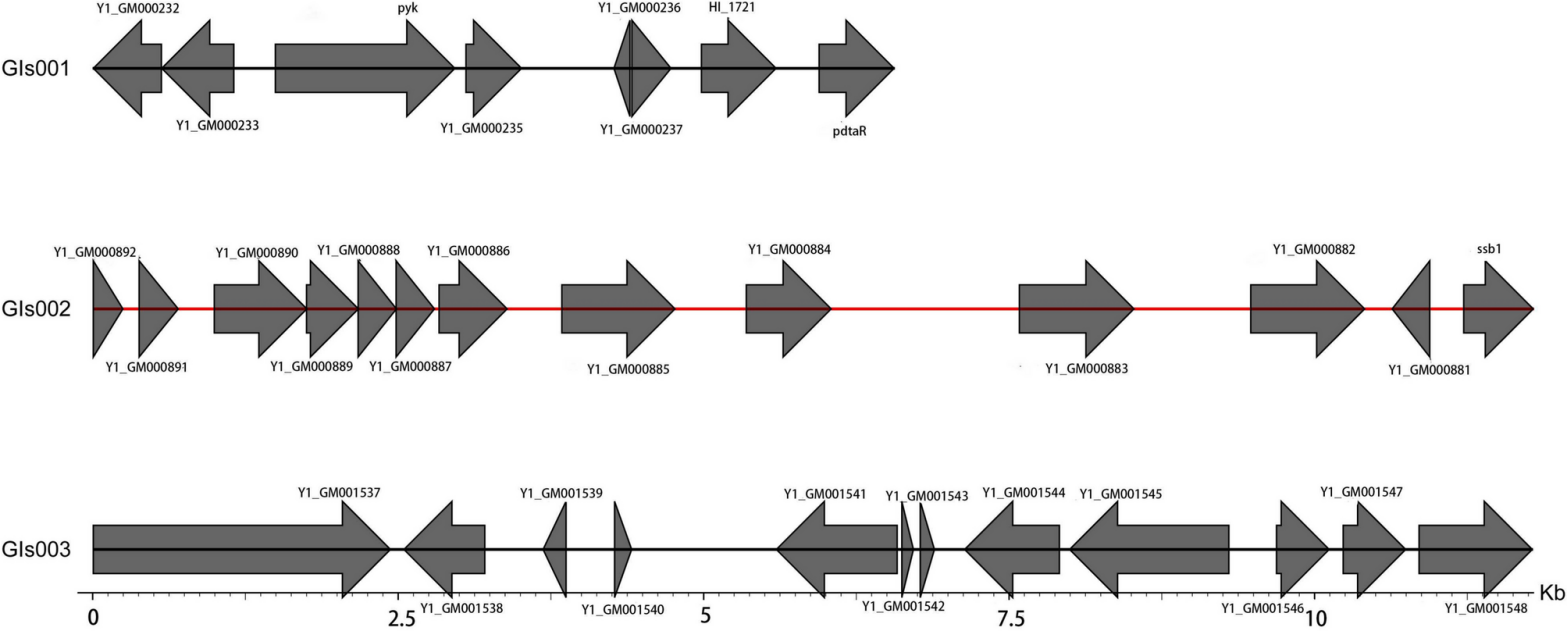
illustrates the gene distribution within the gene island of *Rothia nasimurium* Y1.

**Fig.4:**
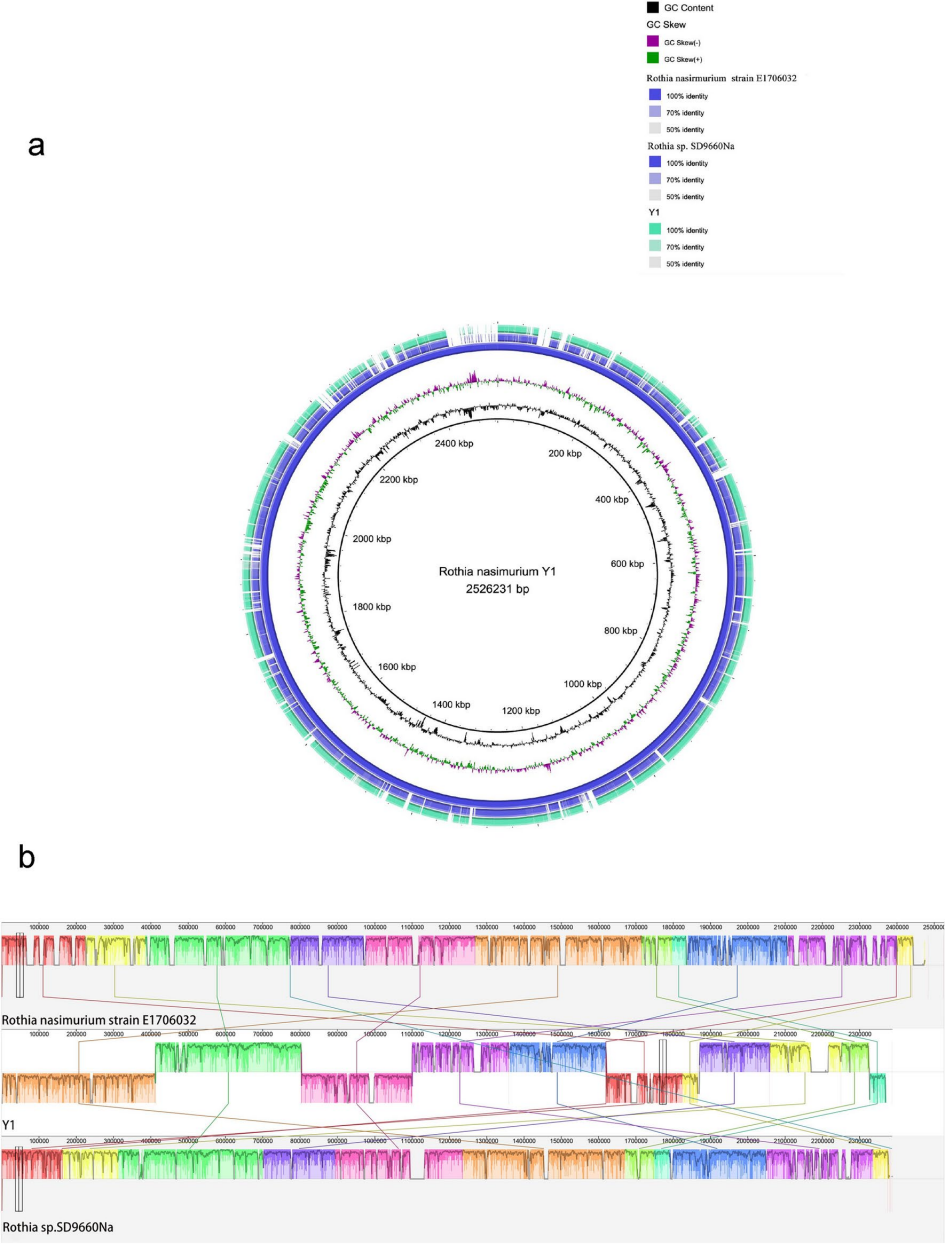
(**a**) Draft genome circle loop of *Rothia nasimurium* Y1, *Rothia nasimurium* strain E1706032, and Rothia sp. SD9660Na were collectively used to construct a circular genome map, The arrangement from the outermost to the innermost sections includes *Rothia nasimurium* Y1 (draft genome sequence), *Rothia nasimurium* strain E1706032 (draft genome sequence), Rothia sp. SD9660Na (draft genome sequence), GC Skew- (GC offset, the inner section represents lower G content compared to C), GC Skew + (GC offset, the outer section represents higher G content compared to C), GC Content, and Genome Sequence Scale (Mbp). (**b**) Y1 covariance analysis with reference strains *Rothia nasimurium* strain E1706032, and Rothia sp. SD9660Na.

Through sequencing analysis of *Rothia nasimurium*, the genome was found to have a total length of 2,387,685 base pairs, with a GC content of 59.35%. Utilizing GeneMarks software(V4.17) (https://genemark.bme.gatech.edu) for prediction and filtering post-sequencing, the total length of the coding genes was determined to be 2,044,335 base pairs. Specifically, the genome featured 3 DNA transposons with a combined length of 175 base pairs, 6 Long Interspersed Nuclear Elements (LINEs) spanning 474 base pairs, and 8 Short Interspersed Nuclear Elements (SINEs) with a total length of 645 base pairs. Additionally, a single retrotransposon was identified, measuring 92 base pairs. The genomic content further included 52 tRNA molecules, collectively covering a length of 3,980 base pairs (supplementary Table 1) . Bacterial genome island is a specific region on the bacterial genome, which can spread various functional genes between bacterial species through horizontal gene transfer, and plays an important role in the process of bacterial survival and host disease. A total of three gene islands GIs001, GIs002, and GIs003 were predicted in the *Y1* bacterial genome (Fig. 3). These islands had a combined length of 30,119 base pairs. By FastANI software (v1.0) ( https://github.com/ParBLiSS/FastANI/releases), the similarity between *Y1* and *Rothia nasimurium* strain E1706032 was 92.28%, and the similarity between *Y1* and *Rothia sp.SD9660Na* was 91.53%. The *Y1* genome sequence was compared with the genome sequences of *Rothia nasimurium strain E170603*2 and *Rothia sp.SD9660Na* to map the genome circle (Fig. 4 a). In the covariance analysis, the Localy Collinear Blocks between *Y1* and the reference strain showed some differences, and some segments were different as well as shifted, suggesting that the degree of differentiation of *Y1* is relatively high, and there may be differences in structure and function (Fig. 4 b). To enhance accessibility to the genomic information, the complete dataset for the strain has been deposited in the National Center for Biotechnology Information (NCBI) under the accession number JAYWIX000000000, facilitated through the upload of the FASTA file.

### Functional annotation of the gene for *Rothia nasimurium*

#### GO database notes

The coding program sequence of *Y1* underwent alignment with the Gene Ontology (GO) database, resulting in the annotation of a total of 1,461 genes. These genes were systematically classified into three principal categories: Cellular Component, Molecular Function, and Biological Process.

**Fig. 5:**
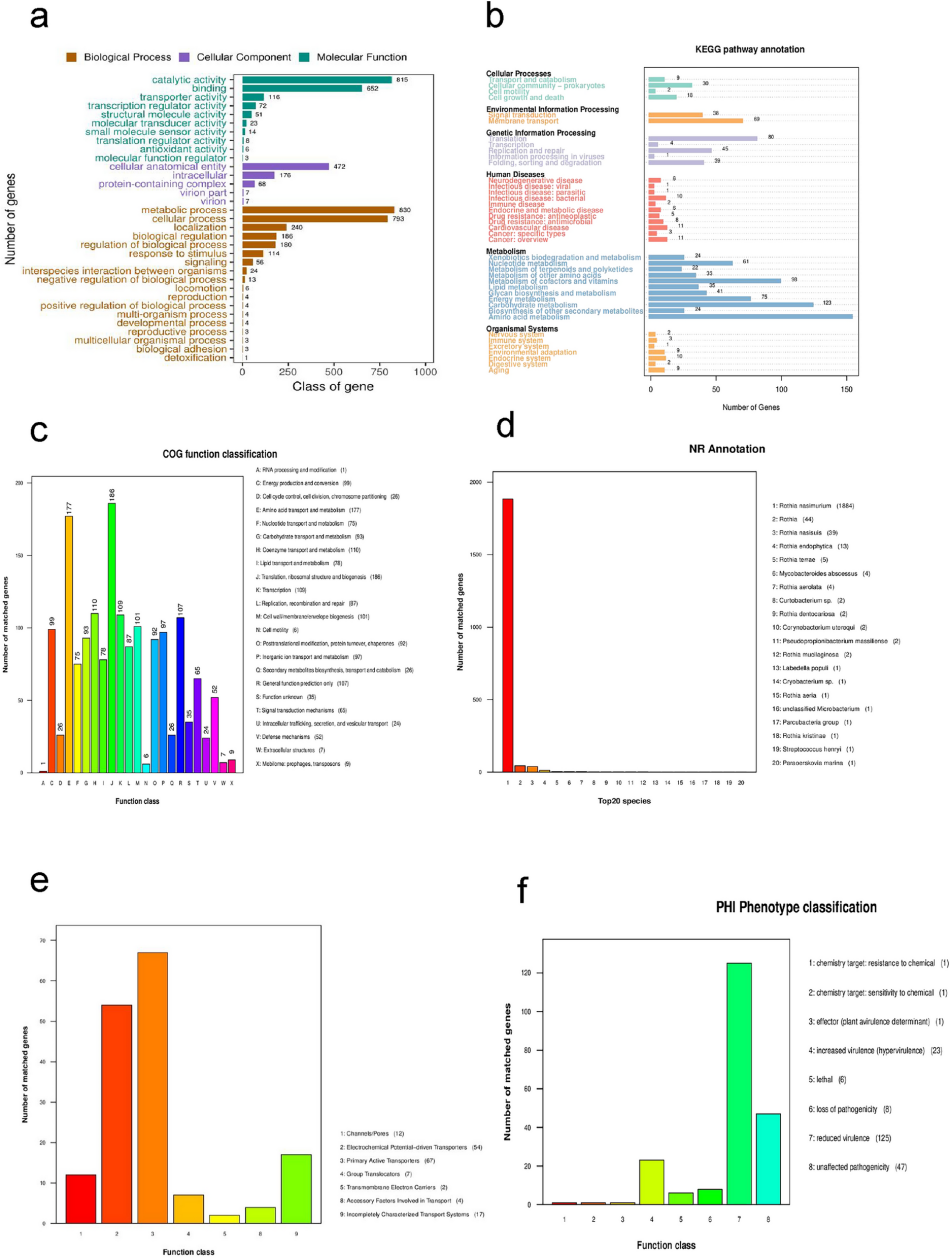
(**a**) presents the GO functional classification map for the Y1 gene of *Rothia nasimurium* Y1, the horizontal axis depicts the GO functional categories in the sample annotation, the right vertical axis indicates the gene count for each annotation, and the left vertical axis expresses the gene count as a percentage of all encoded genes, (**b**) Functional annotation of *Rothia nasimurium* Y1 genes presented in the KEGG metabolic pathway classification map, The numbers indicated on the bars signify the gene count for each annotation. The additional axes provide the codes for each level 1 functional class in the database, with corresponding explanations detailed in the legend, (**c**) Functional annotation map of *Rothia nasimurium* Y1 genes in COG classification. The horizontal axis represents the COG function type, and the vertical axis indicates the gene count for each annotation, (**d**) Functional annotation chart of *Rothia nasimurium* Y1 genes in NR classification, the horizontal axis displays the species ID, and the vertical axis represents the gene count for each annotation. (**e**) Functional annotation chart of *Rothia nasimurium* Y1 genes in TCDB classification, the horizontal axis represents TCDB level 1 classification types, and the vertical axis indicates the gene count for each annotation. (**f**) Distribution of Pathogen PHI Phenotypic Mutation Types, the horizontal axis indicates the type of phenotypic mutation, and the vertical axis indicates the number of genes annotated.

Within the Molecular Function category, the most abundant pathways were associated with catalytic activity and binding. In the Cellular Component category, the most prevalent pathway pertained to cellular anatomical entities. Lastly, in the Biological Processes category, the highest representation was observed in metabolic processes and cellular processes (Fig. 5 a).

#### KEGG database annotation results

Comparing the genome of *Y1* with the KEGG database resulted in the annotation of a total of 1186 coding genes. These genes were distributed across six categories: cell process, environmental information processing, genetic information processing, human disease, metabolism, and biological system, encompassing a total of 40 pathways. Particularly notable were the significant annotations in metabolic pathways related to amino acid metabolism, carbohydrate metabolism, and metabolism of cofactors and vitamins, indicating the bacterium’s robust metabolic capacity^8–10^ (Fig. 5b) .

In addition to its metabolic prowess, *Rothia nasimurium* demonstrated a broad spectrum of drug resistance. The genomic analysis revealed the presence of genes associated with immune regulatory system pathways, contributing to the bacterium’s resilience against various antibiotics (Table 3) .

**Table 3:** Genes associated with drug resistance regulatory system pathways of *Rothia nasimurium.*

Pathway ID	Description	Gene No
map00524	Neomycin, kanamycin and gentamicin biosynthesis	Y1_GM000348
map00525	Acarbose and validamycin biosynthesis	Y1_GM001280, Y1_GM001281
map00521	Streptomycin biosynthesis	Y1_GM000348, Y1_GM001279, Y1_GM001280, Y1_GM001281
map00982	Drug metabolism—cytochrome P450	Y1_GM000229
map00983	Drug metabolism—other enzymes	Y1_GM000152, Y1_GM000416, Y1_GM000662, Y1_GM000663 Y1_GM000666, Y1_GM000706, Y1_GM000851, Y1_GM001349 Y1_GM001350, Y1_GM001718, Y1_GM001794, Y1_GM001881
map00998	Biosynthesis of various antibiotics	Y1_GM000913
map01055	Biosynthesis of vancomycin group antibiotics	Y1_GM001280
map01501	β-lactam resistance	Y1_GM000064
map01502	Vancomycin resistance	Y1_GM000066, Y1_GM000067, Y1_GM000070, Y1_GM000643 Y1_GM000651, Y1_GM000963
map01503	Cationic antimicrobial peptide (CAMP) resistance	Y1_GM001599
map01523	Antifolate resistance	Y1_GM000541, Y1_GM000542, Y1_GM001367, Y1_GM001370
map01524	Platinum drug resistance	Y1_GM001472

#### COG database notes results

The coding sequences of genes in *Y1* were subjected to comparison with the Cluster of Orthologous Groups of proteins (COG) database, resulting in the annotation of 1503 genes with 23 distinct functions. Notably, the most abundant functions were associated with translation, ribosomal structure, and biogenesis, with 186 genes representing 11.19% of the total gene count. Following closely, there were 177 functional genes involved in amino acid transport and metabolism, accounting for 10.65% of the total genes. Additionally, 110 functional genes related to coenzyme transport and metabolism were identified, constituting 6.62% of the total gene count (Fig. 5c). This concurs with the outcomes of the KEGG analysis, affirming the strain’s heightened capabilities in biosynthesis and metabolism functions.

#### NR database notes results

In *Y1*, a total of 2022 genes were annotated using the Non-Redundant Protein Database (NR). The most frequently annotated gene belonged to *Rothia nasimurium*, with 1884 occurrences, followed by *Rothia* with a total of 44 genes (Fig. 5 d) .

#### TCDB annotation results

In the TCDB (Transporter Classification Database) database, a total of 163 genes were annotated for *Y1*. Among these, 67 functional genes were related to primary active transporters, making them the most abundant. Following closely, there were 54 functional genes associated with electrochemical potential-driven transporters. The annotation analysis indicates that primary active transporters serve as the predominant channel proteins in this context (Fig. 5e).

### Analysis of the virulence or pathogenicity of *Rothia nasimurium*

#### Pathogen-Host interaction database (PHI) annotations

Upon comparing the *Y1* genome with the database, a comprehensive annotation revealed a total of 212 genes. Among these, 125 genes were identified as having reduced virulence after mutation. Furthermore, 47 genes remained unaffected by the mutation. Additionally, 23 genes were associated with increased virulence after mutation, while 8 genes were linked to the loss of pathogenicity after mutation. Moreover, 6 genes were found to be associated with lethality post-mutation, and each 1 gene was related to chemotolerance and sensitivity to chemicals after mutation (Fig. 5f).

#### Bacterial pathogenicity virulence factor (VFDB) annotation

Through a comparative analysis of gene sequences from *Y1* against the VFDB database, a total of 112 genes originating from 67 species were identified and annotated. These genes predominantly contribute to various functions such as bacterial adhesion, regulation, nutritional/metabolic factors, hemolysins, immunomodulation, and iron uptake (Table 4). In instances of normal bacterial invasion into the organism, the immune defense system reacts promptly, and phagocytes engulf the bacteria. However, the bacteria manage to evade removal due to the presence of their immunomodulatory proteins. *Rothia nasimurium*, characterized as a conditionally pathogenic bacterium, exhibits hemolysis when co-cultured with pseudointermediate *Staphylococcus aureus*, a phenomenon potentially linked to bacteria hemolysins^5^; *Rothia nasimurium* demonstrates the capability to adhere to host cells, facilitated by its unique and specialized proteins. Moreover, this bacterium has the potential to induce damage to the liver and skin by elevating iron levels in the body, a process mediated by its iron uptake proteins.^11^.

**Table 4:** Statistics on virulence factors of* Rothia nasimurium.*

Function	Virulence factors	Related genes
Adherence	porin	dnaK
	GroEL	groEL2
	GAPDH	plr/gapA
	ClpC	clpC
	Ent	entB
Nutritional/Metabolic factor	Copper exporter	ctpV
	Glutamine synthesis	glnA1
	Iron/manganese transport	sitA
Immune modulation	Capsule	pseF, rfbB, GBS_RS06585, gndA, galU
	HspR	hspR
	Exopolysaccharide	pgi
	GPL locus	rmlA
	Polysaccharide capsule	galE
	AI-2	luxS
	LOS	manB/yhxB
	Pyrimidine biosynthesis	carB
Exoenzyme	Streptococcal enolase	eno
	Zn + + metalloprotease	zmp1
Invasion	LPS	LPG_RS03830
	Petrobactin	GBAA_RS25985, GBAA_RS25990, GBAA_RS25995, GBAA_RS26000
	SigA	sigA/rpoV
Regulation	ClpP	clpP
	Dot/Icm T4SSsecreted effectors	lirB
	EF-Tu	tufA
	PanC/PanD	panC
	PhoP	phoR, phoP
	RelA	relA
Hemolysin	Cytolysin	cylR2
Stress survival	Proteasome-associated proteins	pafA
	Nitrate reductase	narG, narH, narI
	SigH	sigH
Effector delivery system	T6SS-II	clpV
Iron uptake	Mycobactin	mbtI
	IdeR	ideR
	SodA	sodA

### Results of the analysis of drug resistance genes of *Rothia nasimurium Y1*

The draft genome sequencing of strain *Y1* revealed the presence of multiple resistance genes, encompassing categories related to fosfomycin, lipopeptide, mupirocin, glycopeptide, fluoroquinolone, β-lactam, elfamycin, diaminopyrimidine, pyrazinamide, tetracycline, aminoglycoside, lincosamide, Oxadixyl, phenicol, macrolide, sulfonamide, aminocoumarin, and peptide. Additionally, the gene sequence includes several drug-resistant efflux pump genes (Table 5) . These resistance genes play a crucial role in conferring multidrug resistance to *Rothia nasimurium*.

**Table 5:** Statistics of drug-resistant genes in Rothia nasimurium.

Resistant genes category	Resistant genes
fosfomycin	murA
lipopeptide	cls
mupirocin	ileS
glycopeptide	vanA, vanC, vanB, vanE, vanD, vanG, vanF, vanM, vanL, vanO, vanN, mtrA, vanRA, arlR, vanRI, vanRB, vanRC, vanRD, vanRF, vanRG, CpxR, kdpE, vanRM, vanRN, baeR, adeR, vanRL, smeR
fluoroquinolone	gyrA, gyrB, Mfd, mfd, parC
β-lactam	PBP2, PBP2x
elfamycin	EF-Tu
diaminopyrimidine	dfrE
pyrazinamide	pncA
tetracycline	tetB(P), tetQ, tet44, tetT, tetW, tetS, tetM, tetO, otr(A), tet36, tet32
aminoglycoside	mtrA, vanRA, vanRL, arlR, vanRI, vanRB, vanRC, vanRD, vanRE, vanRF, vanRG, kdpE, vanRM, vanRN, baeR, adeR, arlR, baeR, CpxR, smeR
lincosamide	clbC, clbB, clbA, cipA, cfrA, cfrC
Oxadixyl	clbC, clbB, clbA, cipA, cfrA, cfrC
phenicol	clbC, clbB, clbA, cipA, cfrA, cfrC
macrolide	clbC, clbB, clbA, cipA, cfrA, cfrC
sulfonamide	sul3
aminocoumarin	ParY, gyrB
peptide	mtrA, baeR,arlR, CpxR, smeR, vanRA, vanRB, vanRC, vanRD, vanRE, vanRF, vanRG, vanRI, kdpE, vanRM, vanRN
Drug-resistant efflux pump	emrB, farB, tetB(60), tetA(60), tetB(46), novA, tetA(46), lmrD, lmrC, efrB, efrA, msbA, msrB, tcr3, tcmA, otr(B), me, lrfA, QepA2, qepA, tet(43), qacB, qacA, mtrA, vanRA, vanRL, arlR, vanRI, vanRB, vanRC, vanRD, vanRE, vanRF, CpxR, vanRM, vanR,adeR,vanRI,kdpE,vanRN,baeR, smeR

### The results of pathogenicity test in mice

Observation of mice infected with nose drops, within 24 h, the mice appeared depressed, slow movement, appetite loss, after 72 h began to appear hair loss and skin inflammation phenomenon (Supplementary Fig. 2 a and b). The mice did not die during the experiment. No significant lesions were found in the autopsy of mice.

### The results of pathogenicity test in rabbits

Since no significant tissue lesions were found in mice, rabbits were infected with nasal drops to determine whether *Rothia nasimurium* caused organ lesions. After 12 h, the rabbit showed depression, decreased appetite, and slow movement. On the second day, the hair loss and skin inflammation began to appear (Supplementary Fig. 3 a and b). On the third day, the temperature rose to 40.7℃, and the skin ulcer began to appear, serous nasal fluid appears in the nasal cavity. In the autopsy, it can be found that the lungs appear congestion and edema, and the other internal organs are not found obvious lesions (Supplementary Fig. 3 c and d).

## Discussion

The genus *Rothia*, established in 1987 on the recommendation of Georg and Brown, belongs to the family Micrococcaceae^12^. Traditionally, *Rothia* species were considered part of the normal flora in the intestinal tract^13^. However, Bemis et al. conducted research revealing that a strain of *Rothia nasimurium* isolated from dogs exhibited a synergistic hemolytic effect when co-cultured with Staphylococcus intermedius, indicating that *Rothia nasimurium* within the *Rothia* genus can be conditionally pathogenic^5^. Notably, *Rothia nasimurium* has been reported not only to exhibit synergistic hemolysis with* Staphylococcus aureus* but also to display multidrug resistance. This observation was corroborated by the results of the drug sensitivity test conducted in the current study, confirming the multidrug-resistant nature of *Rothia nasimurium*. Given its presence in various animals and potential airborne transmission, there is an implication that the bacterium can be transmitted across species. In this study, draft-genome sequencing of *Rothia nasimurium Y1*, isolated from the peritoneal fluid of diseased sheep, was carried out using second-generation Illumina sequencing techniques

Sequencing results showed that the genome size of *Rothia nasimurium Y1* was 2,387,685 bp, with a GC content of 59.35%, and the length of the encoded gene was 2,044,335 bp. The coding gene sequences were annotated against GO, KEGG, COG, NR,TCDB, PHI and CARD databases to analyze the basic functional features of the genome.

Numerous genes were annotated within the gene island of *Rothia nasimurium Y1*, including SSB1, which is implicated in mediating the recognition of single-strand DNA to ensure gene stability^14^. Additionally, pyruvate kinase (pyk) was identified, serving a pivotal role in catalyzing the irreversible conversion of ADP and phosphoenolpyruvate to ATP and pyruvate. This enzyme plays a significant role in cellular metabolic processes and is associated with carbohydrate and endocrine metabolism, as indicated by information available in the KEGG database^15^. Furthermore, the two-component regulatory system PdtaR/PdtaS was detected, playing a crucial role in the adaptation of Mycobacterium spp. to adverse nutritional conditions, particularly in relation to amino acid metabolism^16^.

The annotation results from the GO database indicated a high enrichment of genes associated with catalytic activity, binding, cellular anatomical entity, metabolic process, and cellular process in the strain. Similarly, in the KEGG database, there was significant enrichment of genes involved in amino acid metabolism, carbohydrate metabolism, and metabolism of cofactors and vitamins. Concurrently, the COG database revealed the highest number of genes encoding functions related to translation, ribosomal structure and biogenesis, amino acid transport and metabolism, and coenzyme transport and metabolism. These findings suggest that the strain likely possesses a robust metabolic and biosynthetic capacity, aligning with the results of annotated gene analysis on the genetic island. This has significant biological implications, considering the critical role of biosynthetic and metabolic processes in bacterial survival.

Moreover, the analysis identified immune-regulated gene pathways encompassing resistance to Neomycin, kanamycin, gentamicin, acarbose, validamycin, streptomycin, vancomycin, β-lactam, antifolate, CAMP, and platinum drugs. This observation aligns well with the multidrug resistance exhibited by *Rothia nasimurium*.

Previous studies have characterized *Rothia nasimurium* as a conditional pathogen. In this investigation, a comparative analysis using PHI annotation and VFDB virulence gene analysis on *Rothia nasimurium Y1* revealed that the predominant virulence factors encompass adhesion and invasion-associated genes, oligosaccharides (LOS), lipopolysaccharides (LPS), capsules, immunomodulation, and iron uptake genes. Notably, dnaK within the genome of *Rothia nasimurium Y1* was identified as a key player in virulence, adhesion, invasiveness, and acid resistance, a role observed in Cronobacter sakazakii^17^. Furthermore, Streptococcus pneumoniae has been demonstrated to release ClpC through autolysis, recruiting it to the bacterial surface. Once on the surface, ClpC binds to and facilitates the activation of fibrinogen, contributing to extracellular matrix degradation and tissue invasion^18^. In a study conducted by I. C. Boels et al., it was observed that galU is responsible for synthesizing diverse complements, leading to bacterial adhesion, motility, and biofilm synthesis. Additionally, galU possesses the capability to prevent the recognition of cell wall-associated complement by complement receptors on phagocytes^19^. The gene galE, associated with galactose metabolism, plays a role in regulating multiple information pathways linked to inflammasome and is crucial for bacterial evasion from inflammasome activation^20^; LuxS/AI-2 system found in the genome, at low cell densities, in the absence of self-inducers, HapR is not produced, so virulence factors are expressed and biofilms are formed. At high cell density, in the presence of self-inducers, HapR is produced and genes that inhibit virulence factor production and biofilm formation are inhibited^21^, LuxS/AI-2 regulates pathogen virulence in multimicrobial communication networks^22^. Additionally, substrates of ClpP have been noted to impact various pathways essential for maintaining normal cellular function. This modulation can influence bacterial virulence, aiding bacteria in evading clearance by the immune system^23^; *Rothia nasimurium* has been labeled a conditional pathogen due to its reported synergistic hemolytic activity with Staphylococcus aureus. This phenomenon may be attributed to the presence of cylR2, a gene that lyses erythrocytes and proves lethal to a broad spectrum of Gram-positive bacteria^24^; Additionally, various iron uptake-related genes (entB, mbtI, ideR, sodA, and sitA) were identified in *Rothia nasimurium*. This finding aligns with Yuhui Kang’s research, which indicated that *Rothia nasimurium* of goose origin induces skin inflammation, hair loss in chicks, and liver damage due to elevated iron concentrations^11^. Moreover, coat shedding and skin inflammation due to elevated iron concentrations have been demonstrated in animal pathogenicity tests. Screening for virulence genes in *Rothia nasimurium Y1* revealed the presence of secondary virulence genes contributing to bacterial invasion. These genes are associated with stress survival, exoenzyme production, and nutritional/metabolic factors.

In previous studies, *Rothia nasimurium* was considered as a conditionally pathogenic bacterium due to its ability to produce synergistic hemolysis with *Staphylococcus aureus*, but this has not been verified in animal studies. In the study by Yuhui Kang and Jiahao Zhang et al^25,26^, it was confirmed that *Rothia nasimurium* could cause coat loss in ducks and geese, but there were no obvious tissue lesions. In the present study, mice were infected with nasal drops and showed coat shedding and skin inflammation, but no obvious tissue lesions. In rabbits infected with nasal drops, there was a marked inflammatory response with elevated body temperature, depression, loss of coat, ulceration of the skin, and plasma nasal discharge. Pathological comprehension, lung congestion, edema and other changes in the lungs indicate that *Rothia nasimurium Y1* is not only a conditionally pathogenic bacterium but also pathogenicity, mainly causing lesions in the respiratory system. In this experiment, *Rothia nasimuriumY1* was isolated from the abdominal fluid of diseased sheep, probably because the lungs were infected at a later stage and the bacteremia produced led to the migration of bacteria into the abdominal fluid. In addition, the distribution of *Rothia nasimurium* to different degrees was also detected in healthy sheep, indicating that *Rothia nasimurium* belongs to the normal bacterial group in the nasal cavity under normal circumstances, and *Y1* in this study was isolated from the abdominal fluid, which has certain pathogenicity to rabbits and mice, and is a highly toxic strain.

In previous studies, *Rothia nasimurium* has been reported as multi-drug resistant. Jiahao Zhang et al. conducted a drug sensitivity test on isolated strains of *Rothia nasimurium* from chickens, revealing resistance to 17 antibiotics, including ciprofloxacin, chloramphenicol, and flufenicol. Sensitivity was observed to varying degrees only to penicillin, amikacin, and tigecycline^27^.

In a study by Miaoli Wang et al., the detection of resistance genes in *Rothia nasimurium* isolated from ducks revealed the presence of resistance genes in β-lactams, aminoglycosides, macrolides, sulfonamides, fluoroquinolones, rifamycin, tetracyclines, lincosamides, and chloramphenicol. The current investigation corroborates these findings by demonstrating that *Rothia nasimurium Y1* exhibits resistance to various antibiotics. Notably, the strain is only susceptible to two drugs: amikacin and vancomycin, not sensitive to cefazolin. This is at variance with the results of the drug sensitivity tests of Zhang et al. and Kang et al^25,26^, probably due to the differentiation of the strains and geographical differences. Furthermore, the genome of *Rothia nasimurium Y1* has been identified to harbor multiple resistance genes. It is noteworthy that intrinsic resistance to phosphomycin can be acquired when bacterial murA is mutated, hindering the entry of phosphomycin into bacteria and impeding the initiation of peptidoglycan synthesis in the bacterial cell wall^28^; In the study conducted by April H Nguyen et al., it was demonstrated that cls activity plays a crucial role in daptomycin resistance in Enterococcus faecalis^29^; The expression of the ileS-2 gene was found to determine the levels of resistance to mupirocin in Staphylococcus aureus^30^; *Rothia nasimurium Y1*'s genome contains multiple fluoroquinolone resistance genes, including gyrA, gyrB, Mfd, and parC. Mutations in gyrA, gyrB, and parC have been linked to bacterial resistance to fluoroquinolones^31,32^. In the research by Jing Han et al., Mfd was identified as essential for the development of fluoroquinolone resistance in bacteria^33^. Bacteria acquire resistance to β-lactam antibiotics through mutations in PBP2 and PBP2Px^34,35^, the resistance of *Y1* to the extended-spectrum cephalosporin ceftriaxone is consistent with findings in Neisseria gonorrhoeae, where a mutation in penicillin-binding protein 2 (PBP2) leads to resistance to ceftriaxone. Nasir Mahmood et al. discovered that the pncA gene is responsible for converting pyrazinamide to its active form, and bacterial resistance to pyrazinamide is acquired through genetic mutations^36^. In the study by Vincent Perreten et al., it was revealed that sul3 gene expression imparts bacterial resistance to sulfonamides. The sul3 gene encodes a 263-amino-acid protein similar to a dihydropteroate synthase found in the 54-kb conjugative plasmid pVP440 from *Escherichia coli*^37^. Elisabeth Schmutz et al. demonstrated that the expression of ParY and gyrB genes expression produces resistance to neomycin versus coumarin, and both genes play essential roles in the mechanism of bacterial resistance to aminocoumarins^38^. Although the genome of *Rothia nasimurium* contains many resistance genes related to aminoglycosides and glycopeptides, the strain remains susceptible to amikacin and vancomycin in drug sensitivity tests. This susceptibility might be attributed to mutations or lack of expression in the relevant resistance genes. Interestingly, the gene pathway associated with vancomycin and vancomycin-like resistance was identified in the KEGG database comparative annotation, despite the strain’s sensitivity to vancomycin. This intriguing observation warrants further in-depth investigation.

## Conclusion

Currently, there is a dearth of research on *Rothia nasimurium*. This study focused on isolating a strain of *Rothia nasimurium*, designated as *Y1*, from the peritoneal fluid of sheep that succumbed to unknown causes on a farm in Xinjiang. The investigation encompassed the isolation, identification, antibiotic susceptibility testing, animal pathogenicity test, draft genome sequencing, and a foundational functional analysis of the genome. Pathogenicity analyses showed that *Rothia nasimurium Y1* contains several virulence factors, including hemolysin and iron uptake genes. Animal tests have shown that it is pathogenic and can cause hair loss, skin inflammation and respiratory system lesions, and more virulent than common strains. The drug sensitivity test disclosed the strain’s resistance to multiple antibiotics, corroborated by the presence of various antibiotic resistance pathways in the KEGG database and an array of resistance genes in the genome sequence. These findings underscore the multidrug resistance of *Rothia nasimurium.* The comprehensive draft genome sequencing and analysis of *Rothia nasimurium Y1* provided insights into its distinctive characteristics, serving as a reference for addressing related bacterial diseases in subsequent clinical practice. The pathogenicity, multi-drug resistance and potential cross-species transmission of this strain need to be paid more attention. Further studies are needed to reveal the mechanism of its synergistic hemolysis and non-expression of certain resistance genes.

## Materials and methods

### Main reagents and instruments

The drug sensitivity tablets were procured from Hangzhou Microbiology Reagent Co. Tryptose Soya Agar (TSA) and Trypticase Soy Broth (TSB), along with the electrophoresis instrument, were acquired from Thermo Fisher Technology (China) Co., LTD. The PCR amplification instrument used was sourced from Bio-Rad, USA, and 2×Taq PCR Master Mix and DL2000 Plus DNA Marker were obtained from Nanjing Nuoweizan Biotechnology Co., LTD. The bacterial genome extraction kit was purchased from Beijing Tiangen Biochemical Technology Co. The NEBNext Ultra DNA Library Preparation kit was purchased from NEB. Female healthy Kunming mice were purchased from Hengchao Biology, New Zealand white rabbits are raised alone in the animal house of the College of Animal Science and Technology of Shihezi University, The diseased sheep were provided by a sheep farm in Shihezi, and consent for this study was obtained from the farm owner. 9 healthy sheep individually bred by Shihezi University. The pathogenic bacteria originated from a sheep farm in Shihezi City within the 8th Division of Xinjiang Production and Construction Corps and are currently archived in the College of Animal Science and Technology at Shihezi University.

### Isolation and purification of bacteria and 16SrRNA sequencing analysis

In this study, two diseased sheep were sent to the laboratory to be tested for the cause of the disease. Diseased sheep were dissected and abdominal fluid was found in the abdominal cavity. The study methods were conducted in accordance with the relevant guidelines and regulations of the ARRIVE guidelines (A2021-7), the Ethics and Research Committee of the College of Animal Science and Technology of Shihezi University approved the experiment (protocol code A2023-0170). The peritoneal fluid from the infected sheep was introduced into Trypticase Soy Broth medium and underwent incubation at 37℃ for 24 hours. Following this, the cultured bacterial solution was applied to Tryptose Soya Agar medium using the four-zone plate scribing method, and the plates were inverted and cultured at 37℃ for 12 hours. Monoclonal colonies were then carefully selected, inoculated into Trypticase Soy Broth medium, and cultured for 6 hours at 37℃ with agitation at 200 revolutions per minute.Add an equal volume of 50% glycerol to the partially purified bacterial solution and store at -20℃. The pure cultured single colonies were coated on glass slides for Gram staining and observed under an oil immersion microscope.

Subsequently, the bacterial fluid underwent DNA extraction using a genomic DNA extraction kit. The extracted DNA was subjected to amplification using the universal primer 27F (5'-AGA GTT TGA TCC TGG CTC AG-3') with 1492R (5'-GGT TAC CTT GTT ACG ACT T-3'). The PCR reaction system consisted of 20 μL, comprising 10μL of 2×Taq PCR MasterMix, 1μL each of the forward and reverse primers, 2μL of the extracted DNA, and 6μL of ddH2O. The PCR reaction program involved an initial step at 95℃ for 5 minutes, followed by 30 cycles of 95℃ for 20 seconds, 57℃ for 30 seconds, and 72℃ for 45 seconds, with a final extension at 72℃ for 7 minutes.

The PCR products were retrieved through 1.5% agar-gel electrophoresis and gel, then sent to Urumqi Youkang Biotechnology Co., Ltd. for 16S sequencing. The sequences obtained from the sequencing of this strain were cut to remove the first and last low-quality fragments, and then BLASTn comparisons were performed in the NCBI database to confirm the strain’s species.

### Drug sensitivity test

In this study, the Kirby-Bauer drug-sensitive disk diffusion method was employed. The following antibiotics were utilized for the assessment of drug sensitivity using this method: amikacin (aminoglycoside), vancomycin (glycopeptide). cefazolin (cephalosporin), norfloxacin (III quinolones), cotrimoxazole (sulfonamide), ampicillin (β-lactam), meropenem (β-lactam), Tetracycline (tetracycline), clindamycin (Lincomycin ), azithromycin (macrolide) and florfenicol (chloromycetin).

The diameters of the bacteriostatic rings produced by each antibiotic were measured and compared against the standards set by the Clinical and Laboratory Standards Institute. This assessment allowed for the determination of drug sensitivity profiles for the isolates against the various antibiotics tested in the study.

### Draft genome sequencing of isolates

Beijing novogene Technology Co., LTD conducted the sequencing of the entire genome of the bacteria. Initially, the isolated and purified bacteria underwent a 12-hour culture at 37℃ in Trypticase Soy Broth medium. Following this, the cultured bacterial solution was subjected to centrifugation at 10,000 RPM for 1 minute. The resulting bacterial precipitation, obtained after centrifugation, was then sent to Beijing novogene Technology Co., LTD for the sequencing process.

### Library construction and computer sequencing

Genomic DNA was extracted using bacterial genome extraction kit, and DNA purity and integrity were detected by agarose gel electrophoresis, and quantified by Qubit. The DNA library was then prepared using the NEBNext Ultra DNA Library Preparation kit after random interruptions.Upon qualifying the DNA samples, the Covaris ultrasonic crusher was employed to randomly fragment the DNA samples. Subsequently, the entire library preparation process was executed, encompassing terminal repair, A-tail addition, ligation adaptor, PCR amplification, fragment screening, and purification steps. This comprehensive procedure resulted in the generation of the final DNA library.

Using Qubit 2.0 preceded library dilution to a concentration of 2 ng/µL and Agilent 2100 (Santa Clara, CA, USA) was employed to detect the insert fragment; qPCR was used for accurate quantification. After qualified, Illumina NovaSeq PE150 sequencing was performed on the different libraries

### Raw data processing

To ensure the precision and dependability of subsequent analyses, the initial sequencing data typically contains a percentage of low-quality information. To address this, readfq (version 10) was employed as the first step in data processing. It aimed to filter and eliminate reads with low-quality bases (mass value ≤ 20) surpassing a specified proportion (default 40%). Additionally, reads with a specific proportion of N-bases (default 10%) were excluded. Furthermore, reads exhibiting an overlap with the adapter beyond a predetermined threshold (default 15bp) were also discarded.

For projects involving small genomes, an additional step was implemented. Samples were compared against a host database to identify and eliminate reads that potentially originated from the host, addressing host contamination concerns. Subsequently, measures were taken to remove duplicate reads, minimizing potential pollution and repetition issues. The outcome of these stringent filtering processes was the extraction of valid and high-quality data, referred to as CleanData, forming the foundation for accurate and reliable subsequent analyses.

### Genome assembly

Following the preprocessing steps to acquire Clean Data, assembly procedures were conducted using SOAPdenovo (version 2.04) (Li R et al., 2010; Li R et al., 2008), SPAdes (Bankevich A et al., 2012), and ABySS (Simpson JT et al., 2009) assembly software. The integration of these assemblies was achieved through the utilization of CISA (Lin S H et al., 2013) software. Subsequently, optimization of the preliminary assembly results and gap filling were performed using gapclose (Version: 1.12) and other relevant software to derive the final assembly outcomes.

To enhance the quality of the assembly, fragments below 500 bp were filtered out, and additional decontamination steps were implemented to address any remaining sample contamination. Subsequent to these refinement processes, evaluations and statistical analyses were conducted, followed by gene prediction on the finalized assembly results. This comprehensive approach ensured the generation of a robust and reliable genomic assembly for further analyses and interpretation.

### Genomic component analysis

Coding gene prediction for newly sequenced genomes utilized GeneMarkS software (V4.17) (https://genemark.bme.gatech.edu) developed by Besemer J et al. in 2001. The identification of interspersed nuclear elements was carried out using Repeat Masker software (Version open-4.0.5) developed by Saha S et al. in 2008. TRF (Tandem Repeats Finder, Version 4.07b), a software developed by Benson G et al. in 1999, was employed for the detection of tandem repeats in DNA sequences. The prediction of tRNA was conducted through tRNA scan-SE software (Lowe T M et al., 1997) in Version 1.3.1.

Gene islands, potentially indicating horizontal gene transfer, were predicted based on sequence composition using Island Path-DIOMB software (Hsiao W et al., 2003) in Version 0.2. This predictive tool relies on phylogenetically biased and mobility genes for the identification of gene islands within genomic sequences. These diverse computational methods collectively contribute to a thorough genomic analysis, aiding in the identification of various genetic elements and features within the sequenced genomes. In addition, based on the genome sequences of other Rochesteria multilocularis strains in NCBI, genome circle maps were drawn using BRIG software and covariance analysis between different strains was produced by mauve software.

### Gene Function annotation

The coding genes were subjected to comparison with various databases, including the Gene Ontology (GO) database (Ashburner M et al., 2000), the Kyoto Encyclopedia of Genes and Genomes (KEGG) database (Kanehisa M et al., 2004; Kanehisa M et al., 2006), the Cluster of Orthologous Groups of proteins (COG) protein database (Galperin MY et al., 2015), the Non-Redundant Protein Database (NR) (Li W et al., 2002), the Transporter Classification Database (TCDB) (Milton SJ et al., 2014), the Pathogen Host Interactions Database (PHI) (Martin U et al., 2015), and the Virulence Factors of Pathogenic Bacteria (VFDB) (Chen L et al., 2012). Diamond software was employed for these comparisons, with the selection of results based on the highest score (default identity ≥ 40%, coverage ≥ 40%) for further analysis and interpretation.

### Phylogenetic tree construction

The construction of a maximum likelihood (ML) phylogenetic tree was carried out using MEGA software(v11,0) (https://www.megasoftware.net) . This tree aimed to depict the evolutionary relationships between the isolated strain and other *Rothia* strains exhibiting high homology in the NCBI database. The phylogenetic inference relied on housekeeping genes specific to *Rothia*. To enhance the robustness of the analysis, a bootstrap value of 1000 iterations was set to assess the reliability of the tree topology.

### Pathogenicity test in mice

*Rothia nasimurium* stored in glycerol was revived in Trypticase Soy Broth medium and cultured until the optical density (OD) value reached approximately 0.6. The concentration of bacterial solution was determinedusing the gradient dilution method. The concentration of bacterial solution was adjusted to 5×10^7^ CFU/mL^39^ by sterile PBS.Ten female healthy Kunming mice were randomly divided into experimental and control groups (5, each). Mice in the experimental group were infected with 50μl *Rothia nasimurium* by nasal drops, with equal drops of left and right nostril, while the control group was injected with equal amounts of sterile PBS by the same method. Each group of mice was kept in a different cage, and their eating, mental state, and mortality rates were systematically monitored throughout the experiment. After 72h , surviving mice were euthanized by CO_2_ asphyxiation, followed by cervical dislocation according to the “Guidelines on the Humane Treatment of Laboratory Animals” (policy no. A2023-017), and the tissues and organs were examined by autopsy to check for pathologies.

### Pathogenicity test in rabbits

After the bacterial recovery, sterile PBS was used to dilute the culture fluid to 5×10^5^ CFU/mL for nasal drip infection^40^. Six female 2-3 kg 3-month-old healthy New Zealand White rabbits were selected, three in each group, and divided into experimental and control groups. The experimental group was treated with 5×10^5^ CFU/mL bacterial solution of 100 μL for nasal infection, and the same amount was dropped on the left and right nostrils. The control group was given 100 μL sterile PBS by nasal drops, and the same amount was dropped into the left and right nostrils. The control group was observed continuously for 7 d, and the clinical symptoms and death were recorded. 7 days later, the surviving rabbits were euthanized by means of deep anesthesia and bleeding in accordance with the “Guidelines for the Humane Treatment of Laboratory Animals” (Policy No. A2023-017), and the tissues and organs were examined by autopsy to check for pathologies.

### Detection of *Rothia nasimurium* in healthy sheep

Design of primers for the detection of *Rothia nasimurium* based on the house keeping gene (GeneBank: CP056080, region: 927582-928451), Y1F : 5'-GCG ACC CAG CAC CAC ATT GAG CAC-3', Y1R: 5'-GAG GAC GGA TGA GGA GTA TGA ACC-3'. It was used to detect the presence of *Rothia nasimurium* in healthy sheep. Since *Rothia nasimurium* was first collected in the nasal cavity, we collected nasal secretions from 9 healthy sheep for testing.

## Supplementary Information

Supplementary Information 1.

Supplementary Information 2.

## Data Availability

The datasets generated during the current study are available in the NCBI repository, [JAYWIX000000000].
