# Supplementary material for: Virulence and resistance gene analysis of Rothia nasimurium by whole gene sequencing
Source: Sci Rep. 2025 Mar 27;15:10583. doi: 10.1038/s41598-025-95405-z (PMC11950441; doi:10.1038/s41598-025-95405-z)
Supplement: Supplementary file 2 — Supplementary Information 2. [file 41598_2025_95405_MOESM2_ESM.pdf]

Supplementary Information

Virulence and Resistance gene analysis of Rothia nasimurium by Whole gene sequencing

Ziyue Lu[1], Sun He[2],Ali Adnan[1], Wenyu Fan[1], Jinliang Sheng[1], Yanming Sun[1], Yanbing Zhang[1] * and Gang Wang[2]*

1.College of Animal Science and Technology, Shihezi University, Shihezi, Xinjiang, China
2.TECON Pharmaceutical Co.,Ltd, Urumqi, 830011, China
* Correspondence:
Yanbing Zhang, College of Animal Science and Technology, Shihezi University, E-mail: zhangyanbing@shzu.edu.cn;

Supplementary Table

Supplementary Table 1

Supplementary Figures

Supplementary Fig .1

Supplementary Fig .2

Supplementary Fig .3

Supplementary Fig .4

Supplementary Fig .5

Supplementary Table 1 Genome statistics of *Rothia nasimurium*

Items	Length/bp	Amount
Gene	2 387 685	
GC content/%	59.35	
Coding gene	2 044 355	2119
DNA Transposon	175	3
Long interspersed nuclear elements	474	6
Short interspersed nuclear elements	645	8
Rolling circle	92	1
tRNA	3980	52
Gene island	30 119	3

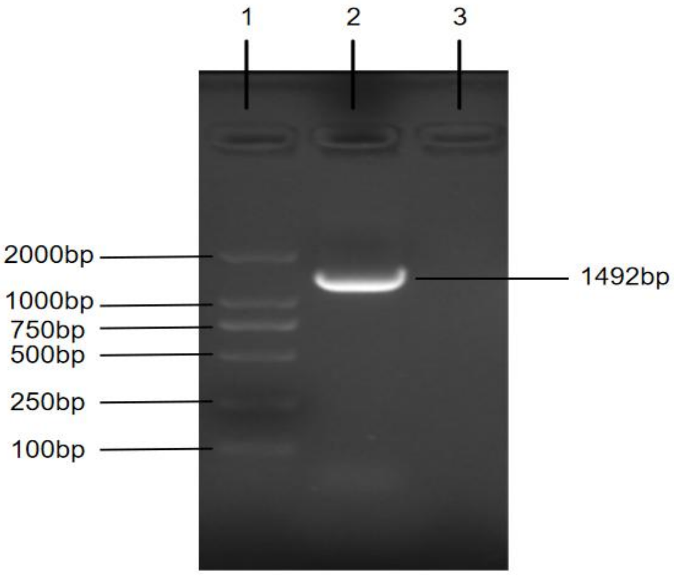

Supplementary Fig .1 **Results of 16SrRNA agarose gel electrophoresis**
1.DL2000 Plus DNA Marker； 2. 16SrRNA gene PCR amplification product；
3. Negative control hole.
In the second lane, a distinct band of approximately 1492 bp size is observed.

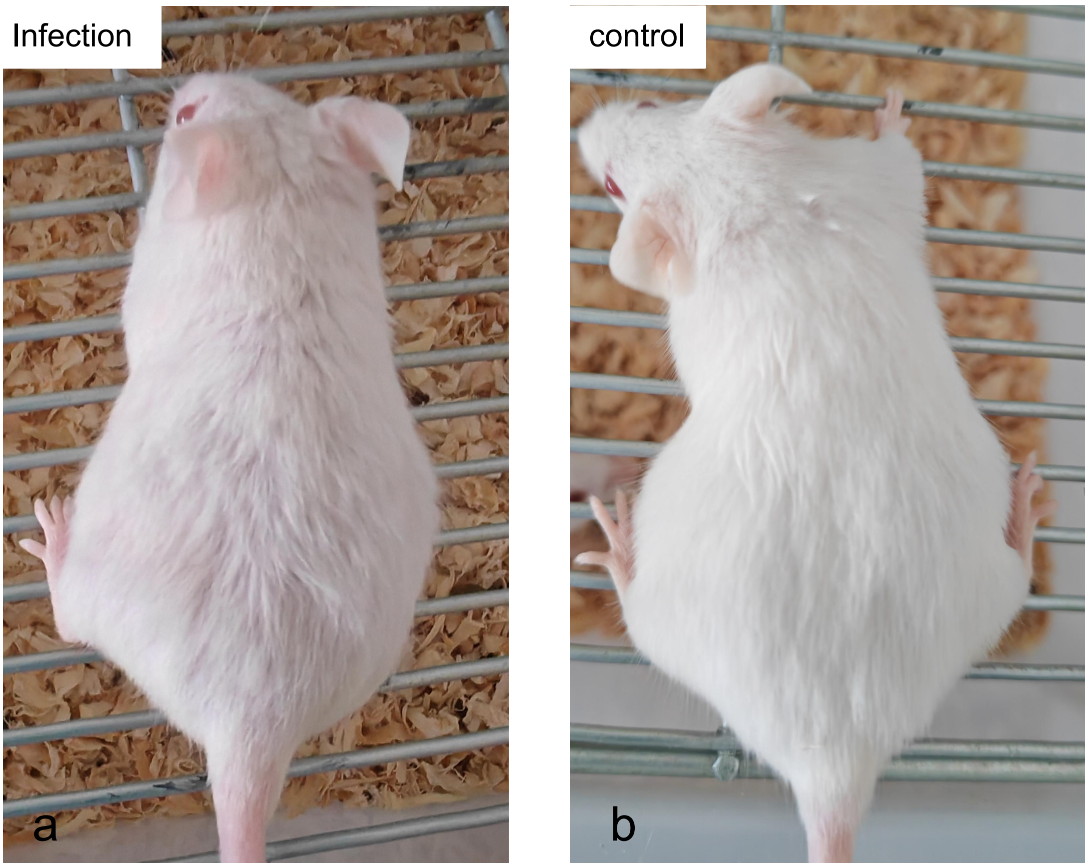

Supplementary Fig .2 **Pathogenicity test in mice**

（a） Experimental group of mice with sparse coat and red, swollen skin

（b） Control mice with normal coat and skin

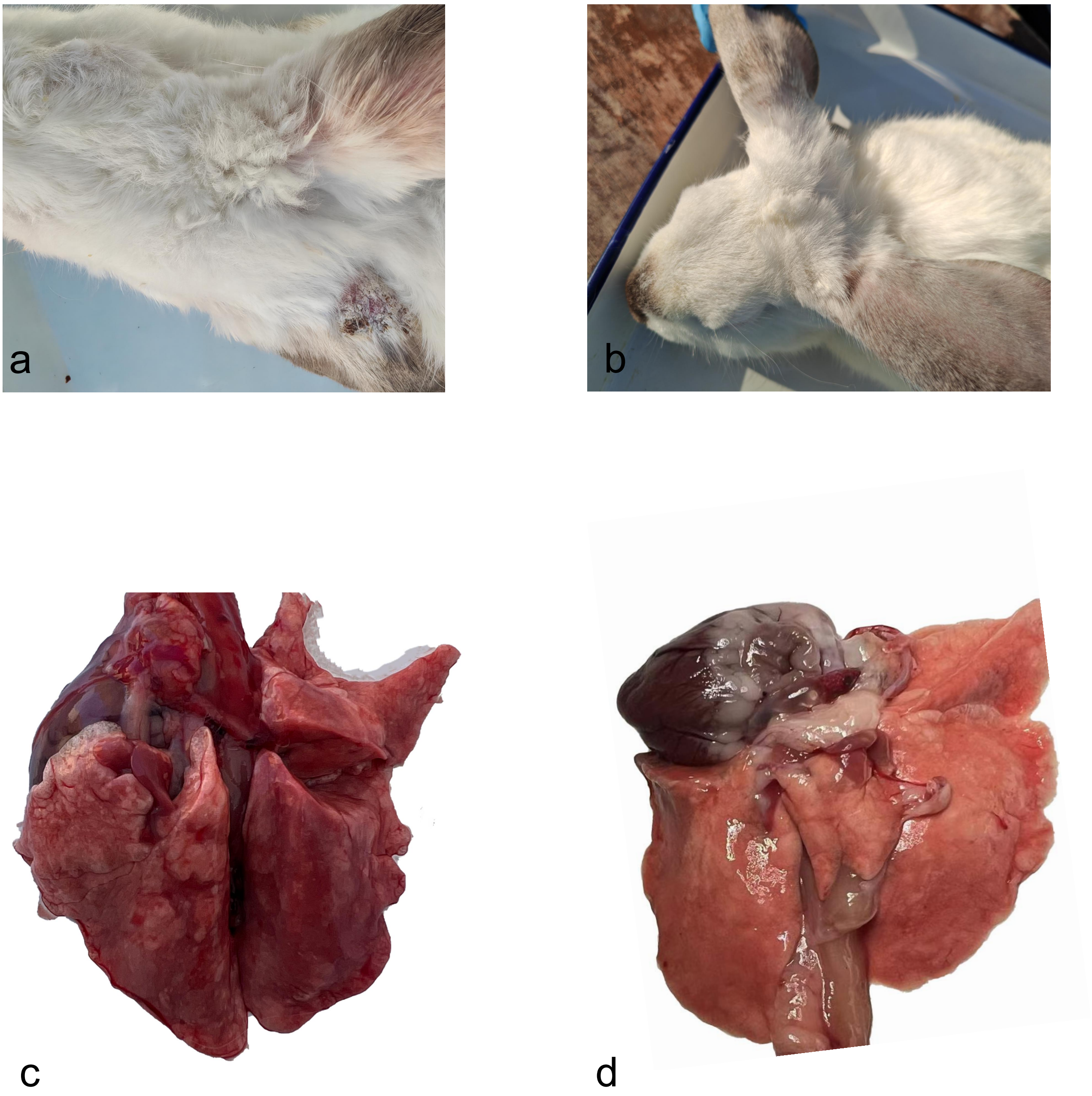

Supplementary Fig .3 **Pathogenicity test in rabbits**

（a） The rabbits in the experimental group had scanty and disorganized coats, and showed severe hair loss at the base of the ears and dermatologic signs of dander with ulcers.

（b） The rabbits in control group had normal fur and skin

（c） The rabbits in the experimental group had pulmonary congestion and edema

（d） The rabbits in the control group had normal lungs

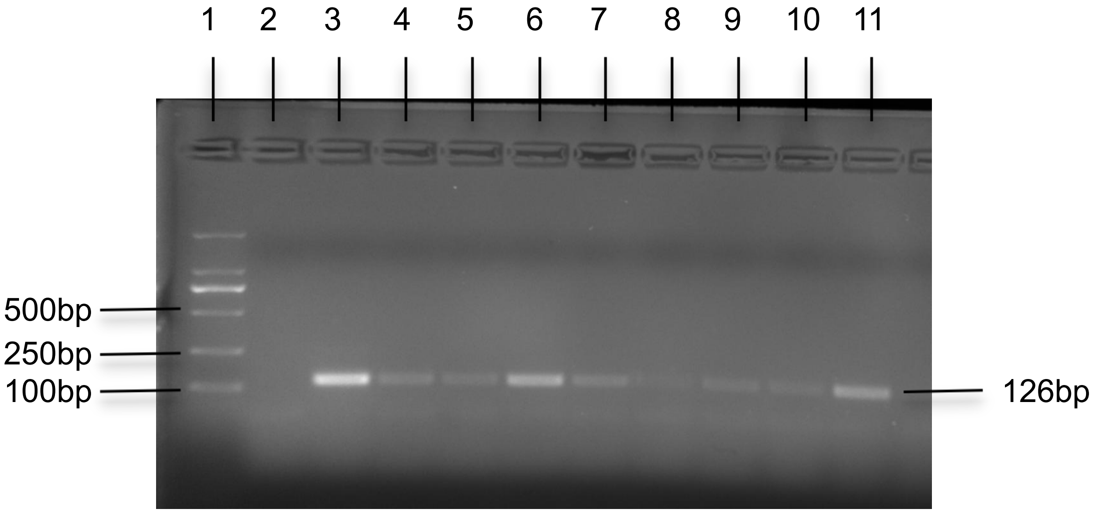

Supplementary Fig .4 **Results of Healthy Sheep Testing**
1.DL2000 Plus DNA Marker；
2. Negative control hole；
3-11. Healthy Sheep
A band of size 126 bp can be found

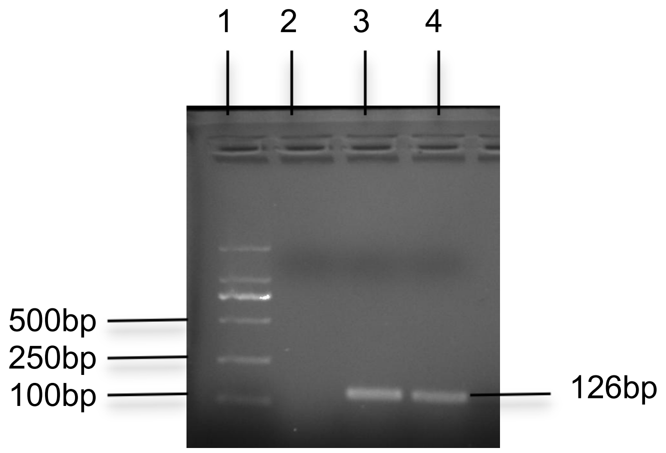

Supplementary Fig .5 **Detection of primer test**
1.DL2000 Plus DNA Marker；
2. Negative control hole.；
3-4. Y1
A band of size 126 bp can be found
